# Tree-Ring Based May-July Temperature Reconstruction Since AD 1630 on the Western Loess Plateau, China

**DOI:** 10.1371/journal.pone.0093504

**Published:** 2014-04-01

**Authors:** Huiming Song, Yu Liu, Qiang Li, Na Gao, Yongyong Ma, Yanhua Zhang

**Affiliations:** 1 The State Key Laboratory of Loess and Quaternary Geology, Institute of Earth Environment, Chinese Academy of Sciences, Xi’an, China; 2 Department of Environmental Science and Technology, School of Human Settlements and Civil Engineering, Xi’an Jiaotong University, Xi’an, China; 3 University of Chinese Academy of Sciences, Beijing, China; 4 Institute of Geology, China Earthquake Administration, Beijing, China; Chinese Academy of Sciences, China

## Abstract

Tree-ring samples from Chinese Pine (*Pinus tabulaeformis* Carr.) collected at Mt. Shimen on the western Loess Plateau, China, were used to reconstruct the mean May–July temperature during AD 1630–2011. The regression model explained 48% of the adjusted variance in the instrumentally observed mean May–July temperature. The reconstruction revealed significant temperature variations at interannual to decadal scales. Cool periods observed in the reconstruction coincided with reduced solar activities. The reconstructed temperature matched well with two other tree-ring based temperature reconstructions conducted on the northern slope of the Qinling Mountains (on the southern margin of the Loess Plateau of China) for both annual and decadal scales. In addition, this study agreed well with several series derived from different proxies. This reconstruction improves upon the sparse network of high-resolution paleoclimatic records for the western Loess Plateau, China.

## Introduction

Recently, the impacts of global warming on different regions especially on environmental fragile regions have received much attention [1]–[3]. The Loess Plateau of China, located at 100°54′–114°43′E and 33°43′–41°16′N, is one of the most climate-vegetation sensitive regions in China [4]. The study showed that extreme temperature events became more severe and frequent [5], which have significantly affected both the social economy and the people living in this area. Thus, there is a need to understand the mechanisms of climate change on the Loess Plateau. For this purpose, recent climate change must be studied in the context of the past one hundred to one thousand years. Instrumental weather records only provide limited data for approximately the last 60 years in China and are inadequate for examining the low-frequency variability that may underlie short-term climatic trends [6]. Tree-ring records are an important resource for understanding past climate change and can provide useful climate information for several centuries or even millennia beyond the instrumental record. Several dendroclimatological studies have been performed on the Loess Plateau in recent decades. Tree-ring based precipitation/drought reconstructions have been developed for the western Loess Plateau [7]–[10], and temperature reconstructions have been reported for the eastern and central Loess Plateau [11]–[13]. Nevertheless, temperature reconstructions remain limited for the western Loess Plateau.

The goals of this study were to reconstruct a seasonal temperature over the past 400 years using tree-ring widths from Mt. Shimen (MSM) and investigate the temperature variability at decadal to multi-decadal scale on the western Loess Plateau. Basing on the temperature reconstruction, the following questions need to be solved: (i) explore the relationship between seasonal temperature and drought events on the western Loess Plateau, (ii) find the affecting factor for the temperature variation.

## Materials and Methods

### Study area and climate data

**Figure 1:**
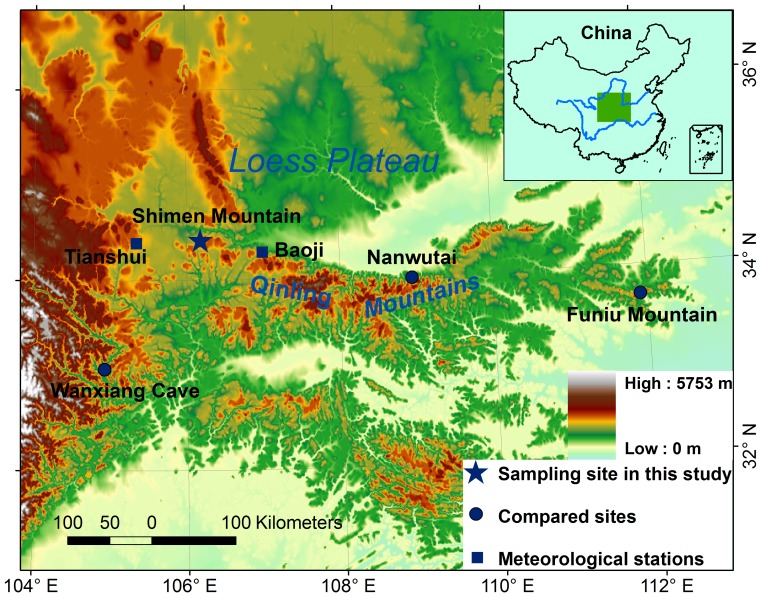
Map of the sampling site.

Mt. Shimen (Fig. 1) is located on the northern slope of the western Qinling Mountains (QLM) and adjoins the Loess Plateau in the north. The climate, water resources and vegetation differ between the northern and southern slopes of the QLM due to its high altitude. The northern slopes are cold and dry, and the southern slopes are warm and humid. Therefore, the QLM is the transitional area between northern and southern China. The climate system for the study area is complicated due to its unique geography; the climate is arid to semiarid continental and is affected by the East Asia summer monsoon, the Indian monsoon and the Tibetan Plateau monsoon [14]. During the summer, the East Asia summer monsoon is the predominant factor and brings large quantities of heat and water vapor. During the winter, the weather throughout the entire Loess Plateau is controlled by the Mongolian High, which brings cold, dry air masses into the area [14].

**Figure 2:**
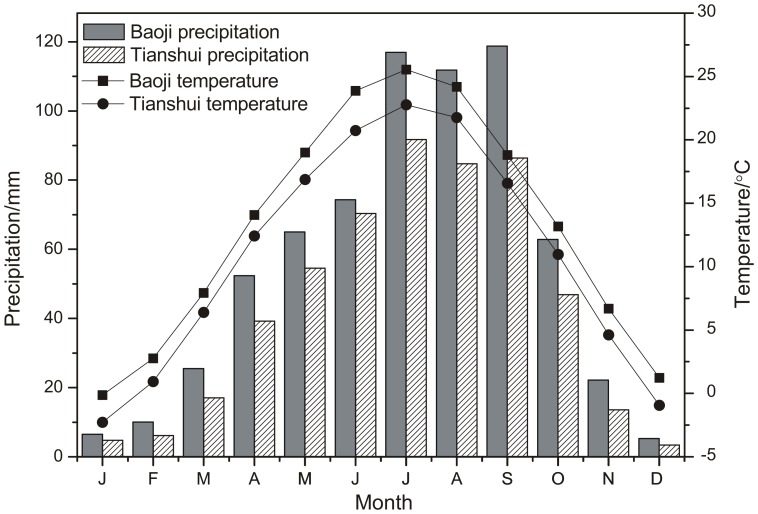
Average monthly temperature and precipitation for the Baoji (AD 1952–2011) and Tianshui (AD 1951–2003) meteorological stations.

The closest meteorological stations to the sampling site are at Tianshui (34°21′N, 105°27′E, 1141.7 m a.s.l., from AD 1951 to 2003) and at Baoji (34°12′N, 107°05′E, 612.4 m a.s.l., from AD 1952 to 2011). The Baoji station, which provides a relatively long record of observed data spanning 60 years, was chosen to investigate the relationships between tree-ring width and climate. The monthly mean temperature and total precipitation for both stations are presented in Figure 2. The Baoji station had more precipitation and higher temperature compared to the Tianshui station. However, both stations showed similar temperature and precipitation variation trends and showed the highest amount of rainfall from July to September. According to the records from the Baoji station, the mean annual precipitation from AD 1952 to 2011 was 675.18 mm (Fig. 2), of which 52.68% occurred from July to September (Fig. 2). July (mean temperature of 25.59°C) and January (–0.15°C) were the warmest and the coldest months, respectively. The Palmer Drought Severity Index (PDSI) was obtained from the global PDSI data set developed by Dai et al. [15]. We used the PDSI data from the nearest grid point (33.75°N, 106.25°E).

### Sampling site and chronologies

With the permission of local forest authority, we collected living *Pinus tabulaeformis* Carr. samples from the top of MSM (34°27′N, 106°09′E) at elevations ranging from 2050 to 2150 m (Fig. 1). The sampling site was located on steep rocky slopes having poorly developed soil, sparse ground cover and a low density of trees. Samples were fine-sanded and crossdated using standard dendrochronologial techniques [16]. The accuracy of the tree-ring dating and measurements were then checked and confirmed using the quality-control program COFECHA [17]. Cores with ambiguous results and those that were too short were excluded from further analysis; 64 cores from 34 trees were used to establish the chronology.

**Figure 3:**
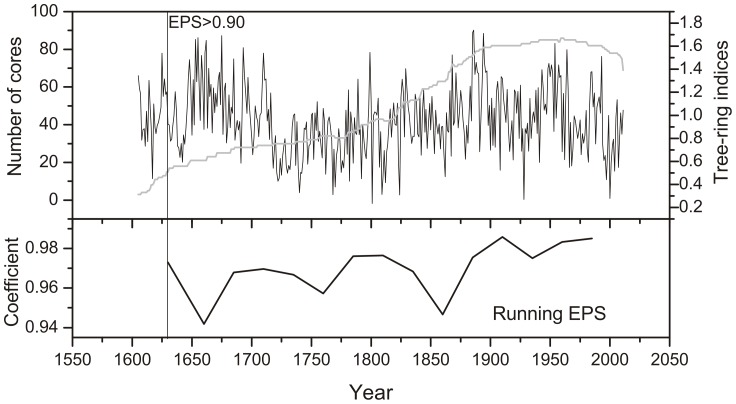
MSM tree-ring STD chronology, running EPS and sample size.

Measured ring-width series were standardized to a tree-ring chronology using the program ARSTAN. (http://www.ldeo.columbia.edu/res/fac/trl/public/publicSoftware.html) [18]. During this process, age-related trends were removed by fitting a negative exponential curve or straight line function to the data. The individual index series were then combined into a single chronology by calculating a bi-weight robust means [18]. All subsequent analyses used the “standard chronology”, which conserves more low frequency signals than other chronologies. The expressed population signal (EPS) was used to evaluate the reliability of the tree-ring chronology. Values exceeding 0.85 were considered acceptable [19]. The values of the running EPS from AD 1630 to 2011 were greater than 0.9 (Fig. 3), which affirmed the reliability of the chronology [18]. The series before AD 1630 could be regarded as reference (with 7 trees and 13 cores).

### Method

To identify climate-growth relationships for the Chinese pine on MSM, a correlation analysis was performed for monthly mean temperature, monthly total precipitation and PDSI over the previous and current years of tree growth. The model stability and reliability were assessed using a split-sample method in which the model was divided into two subsets of equal length [20], [21].The Pearson correlation coefficient (*r*), the positive RE (reduction of error) and CE (coefficient of efficiency) are the items to evaluate the results. Spatial correlations were used to assess the representativeness of our reconstruction by using the KNMI Climate Explorer (Royal Netherlands Meteorological Institute; http://climexp.knmi.nl).

## Results and Discussion

### Relationships between tree-ring widths and climate data

**Figure 4:**
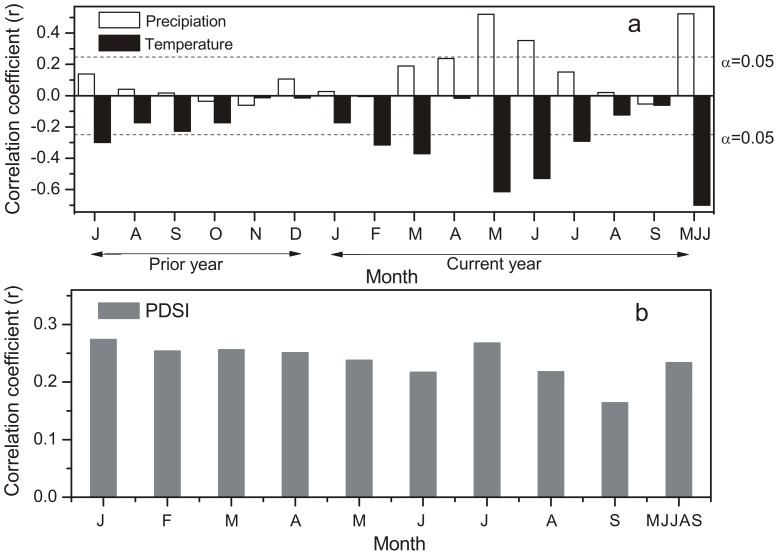
Correlations between the tree-ring STD chronology and monthly mean temperature (AD 1952–2011), monthly total precipitation (AD 1952–2011) and monthly mean PDSI (AD 1952–2005). Horizontal dashed lines represent the 95% confidence level.

Correlation analysis showed that the ring-width STD chronology was negatively correlated with temperature for all months (Figure 4a). In contrast, the correlations between precipitation and tree growth were positive and slightly lower than those for temperature. This means that cool and wet conditions favor the growth Chinese pine in the study region. May-July precipitation and temperature are both significantly correlated with STD chronology, suggesting that May-July is a crucial period for the growth of Chinese pine on MSM. Negative correlations between tree-ring widths and temperature and positive correlations with precipitation were reported in other tree-ring studies on Loess Plateau [8], [9], [11], [12], which suggests that the soil moisture regime during the onset of the growing season is important for tree’s growth on the Loess Plateau. During May to July when the precipitation is not enough for tree’s growth, high temperatures and strong radiation input intensify evaporation rates and then decrease moisture content in the topsoil further. The growth of fine roots gets inhibited and the uptake of nutrients might be hampered [22]. This moisture stress will lead to narrow or missing rings. Another study (Mt. Kongtong, near this study site) showed that the temperature played more important role in the moisture conditions (PDSI) than precipitation [23]. It has been observed that: (i) precipitation anomalies tend to dominate the change of PDSI in cold season when evaporation is minimal; (ii) the effect of temperature on PDSI becomes more important in warm seasons [24]. In addition, other studies showed that tree rings are significantly correlated with PDSI [8], [9], [25], whereas trees rings on MSM showed a weak correlation with PDSI (Fig. 4b), which is different from other study results. The strongest correlations were found between annual ring-width indices and May-July temperatures from AD 1952–2011 (r = –0.70). Basing on the analysis above, it makes sense that May-July mean temperature is the limiting factor for tree growth on MSM. The same response was observed in Nanwutai [11] and the Funiu Mountains [26], which are also located on the northern slope of the QLM.

### May-July temperature reconstruction for Mt. Shimen

**Figure 5:**
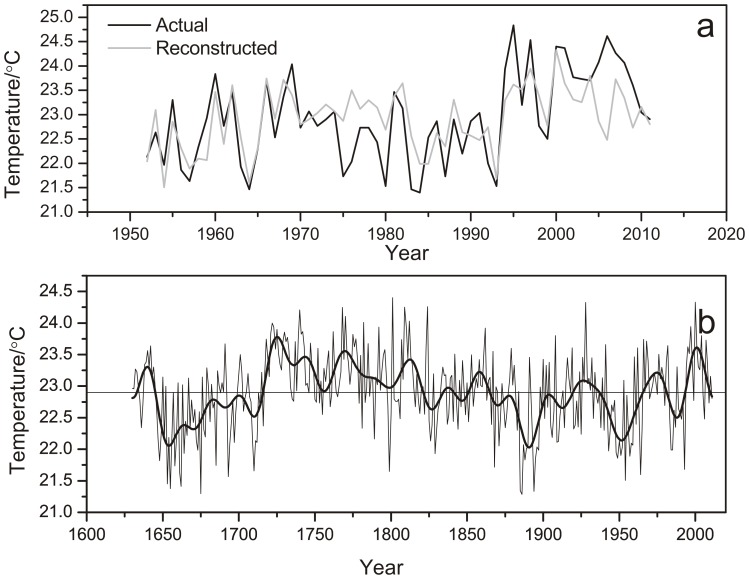
Comparison between observed and reconstructed mean temperatures from May to July . (a) The reconstructed (gray line) and observed (black line)temperature from May to July. (b)The reconstructed May–July mean temperatures after 20-year low pass filter for MSM since 1630.

Based on the computational analysis presented above, the temperature from May–July was reconstructed from MSM tree rings using the linear regression model as follows:
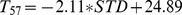


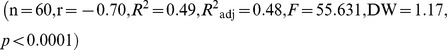



where *T*_57_ is the temperature from May to July (AD 1963–2011), and *STD* represents the standardized tree-ring chronology. The calibration and verification statistics are presented in Table 1. Correlation coefficients for the split-sample validation periods showed a strong relationship. The positive RE and CE results indicated that the reconstruction contributes unique paleoclimatic information. In addition, Figure 5a showed that the reconstruction closely tracked the observed temperature.

**Table 1:** Statistics for a split calibration-verification procedure (*p*<0.01).

Calibration				Verification			
Period	*r*	*R* ^2^	*R* ^2^_adj_	Period	*r*	RE	CE
1952–1981	−0.69	0.47	0.45	1982-2011	−0.71	0.46	0.29
1982–2011	−0.71	0.51	0.49	1952–1981	−0.69	0.48	0.15
1952–2011	−0.70	0.49	0.48	–	–	–	–

### Temperatures on Mt. Shimen

Figure 5b shows the reconstructed May-July temperature variations since AD 1630 and its 20-year low-pass filter on MSM. This series shows interannual to multi-decadal variability (Fig. 5b). The long cooling period from AD 1640 to 1720 corresponds with well-known periods of solar minima (Maunder Minimum) during AD 1645–1715 [19]. Two obvious cool periods that occurred during the 1880s–1900s and the 1930s–1960s coincide with the 1900 minimum (AD 1880–1900) and a slight decrease in sun activity from AD 1940–1970, respectively [27]. The cool period during the reconstruction matched all solar minima except the Dalton minimum (AD 1800–1820), implying that solar forcing likely played an important role in past climate change for MSM. The longest warming trend occurred during AD 1720s–1810s and was followed by a mild climate for approximately 50 years. In addition, the filtered reconstruction indicates that the warming since AD 1970s is not significant and does not exceed the natural temperature variations that occurred over the past 400 years. AD 1928 stands out as an unusually warm year and corresponds with the drought event in AD 1928–1929, which has been noted as a dominant feature of the drought record throughout northern China [28], [29]. Additionally, the warm year of AD 2000 is consistent with the severe drought that occurred in China [9].

### Comparison with other tree-ring based temperature reconstructions

**Figure 6:**
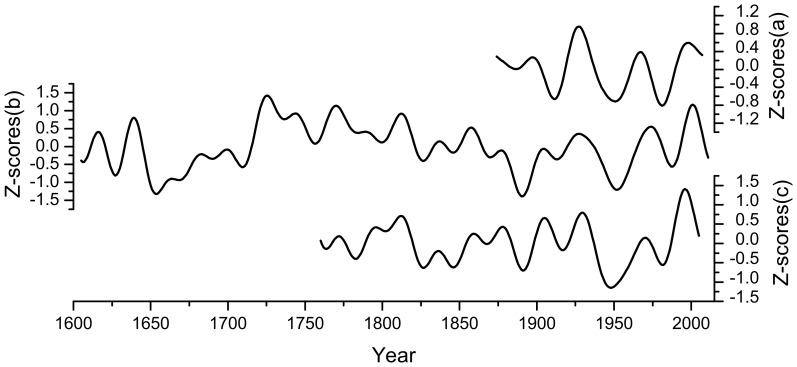
Tree-ring based temperature comparison among three sites from the northern slope of Qinling Mountains. (a)FNM [26], (b) MSM (this study) and (c) NWT [11].

To evaluate the reliability of the reconstruction, we compared the reconstruction with two other tree-ring based temperature reconstructions from Nanwutai (NWT) [11] and the Funiu Mountains (FNM) [26]. MSM, NWT and FNM are all located on the northern slope of the QLM (Fig. 1). The Chinese pines from these three sites synchronously respond to May-July temperature variations, indicating that the growth patterns of trees throughout the region capture large-scale variations in the growing season due to the barrier function of the QLM. In addition to having the same growth patterns, the May-July temperature from MSM displays similar patterns of variation at high- and low-frequency to those in the NWT and FNM temperature records (Fig. 6). The temperature of MSM is highly correlated with those of NWT and FNM with r = 0.49 (n = 246, *p*<0.0001) and r = 0.32 (n = 132, *p*<0.0001) inter-annually and r = 0.49 and r = 0.60 after 20-year low-pass filtering, respectively. The most prominent and synchronous feature of the three reconstructions is the occurrence of cooler temperatures during the AD 1930s–1960s. The coherent temperature variation from the three sites indicates that the May–July temperature for the Northern slope of QLM has been consistent from east to the west during the last several centuries. In contrast, the tree-ring based temperature from Western Sichuan ([30] to the south of QLM) is significantly different because of the climate division function of QLM.

### Spatial representativeness and comparisons with other records

**Figure 7:**
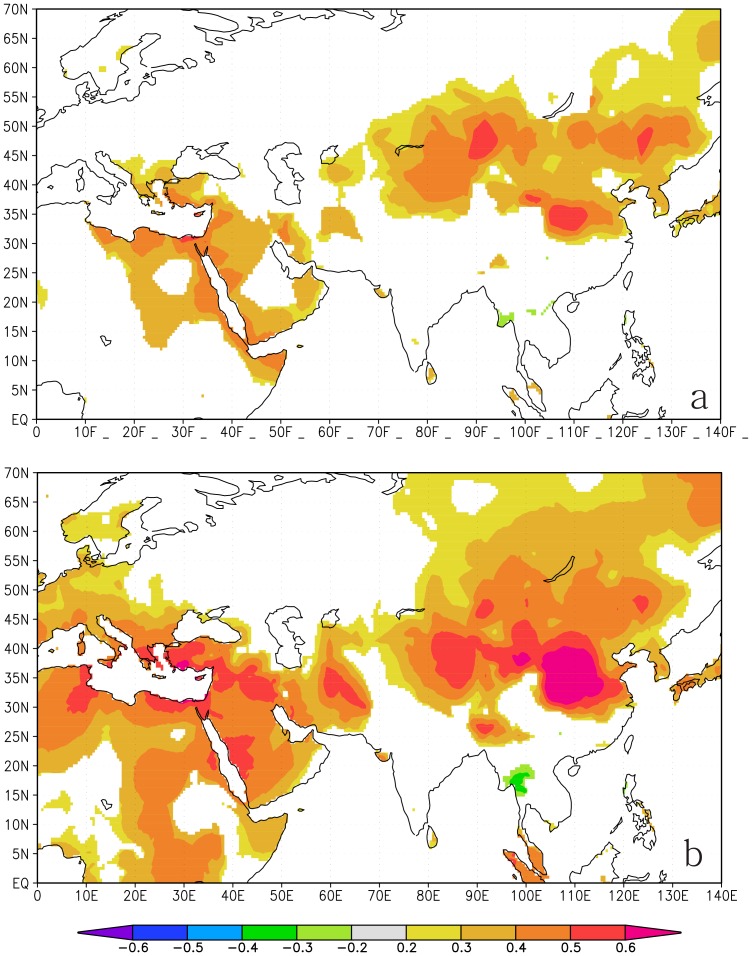
Patterns of field correlation in our study . (a) Correlations between the reconstructed temperatures with the CRU TS3 temperature during May–July (AD 1952–2009). (b) Correlations between the observed temperatures with the CRU TS3 temperature during May–July (AD 1952–2009).

Spatial correlations were computed between our temperature reconstruction, meteorological records and CRU TS3 temperature datasets over the calibration period (AD 1952–2009). The reconstruction and observational data showed a similar correlation pattern (Fig. 7), and they all correlated significantly with temperatures in north-central China; therefore, the MSM temperature reconstruction could represent the temperature variability for northern China to a certain extent. Additionally, the temperatures for the coastal regions of the Mediterranean and the Red Sea correlated significantly with the MSM temperature and the Baoji temperature during the period from May to July. The mechanism of this correlation between these geographically separated areas is complicated and will be explored in future studies.

**Figure 8:**
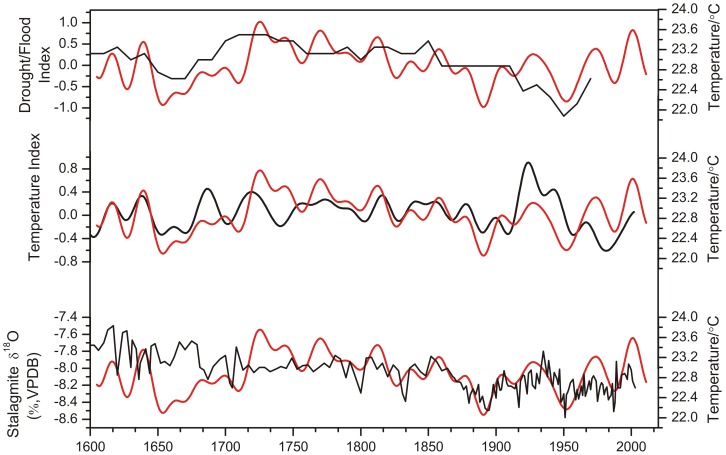
Comparison between the MSM temperatures (red) and other series (black). (a) Historical documents based on the Longxi Drought index [31]. (b) Regional temperature reconstruction for northern China [32] and (c) δ^18^O time series of stalagmites from Wanxiang Cave, China [33].

The series derived from the different proxies in the surrounding area provide the opportunity to validate our reconstruction and help us understand various aspects of the climate variation pattern for the western Loess Plateau. Figure 8 shows the comparison between the May–July temperature and series based on stalagmites, historical documents and multi-proxies. The variation trends between the MSM temperature and historical documents based on the Longxi Drought index [31] are highly similar(Fig. 8a), indicating that the drought and temperature patterns for the western Loess Plateau are synchronous with the long-term trend. The good agreement with regional temperature reconstruction for northern China [32] indicates that the MSM temperature reconstruction successfully captures the temperature variability for the large-scale region (Fig. 8b). The stalagmite record from Wanxiang Cave, China, characterizes the Asian Monsoon history over the past 1,810 years [33]. The May–July temperature for MSM maintained a high level of similarity to the stalagmite record since AD 1750, especially during the cool periods of AD 1880s–1900s and AD 1930s–1960s (Fig. 8c). Thus, the Asian Monsoon may influence the temperature variation of the MSM region to a certain extent at the low frequency variation. The study showed that Summer Asian Monsoon precipitation is driven by the thermal contrast between Asia and tropical Indo-Pacific and therefore should be linked to regional temperature change [33]. The 11-year running correlation analysis indicates that there is a positive correlation between the East Asian Summer Monsoon Index and summer surface air temperature in eastern China during 2/3 of the time span of 1880–2004, implying the impact of subtropical land-sea thermal contrast on the East Asian Summer Monsoon Index [34]. Although the monsoon series indicated by the stalagmite record was not weak during the period AD 1640–1720, the other three series show common decreased variations during the same period, which is consistent with the Maunder Minimum.

## Conclusions

Here we described a reconstruction of the mean May–July temperatures for MSM on the western Loess Plateau based on tree-ring width data dating back to AD 1630. This reconstruction accounts for 49% of the actual temperature variance over the period from AD 1951 to 2011 (48% after adjustment for the loss of degrees of freedom). The reliability of our reconstruction was confirmed by comparing it with other records from nearby areas. The similarity among the tree-ring based temperature reconstructions obtained from the northern slope of the QLM reflects large-scale temperature variations over the past several centuries. The reconstruction compared well with series derived from other proxies, indicating that the drought and temperature variations were synchronous with the long-term trends on the western Loess Plateau. The reconstruction displayed cooler temperatures during periods of known solar minima except during the Dalton minimum. The East Asian summer monsoon may influence the temperature variation at MSM to some extent. However, additional research is needed to develop a tree-ring network that can reflect spatial climate variations on the Loess Plateau.
